# Supplementary material for: Cardiometabolic outcomes of women exposed to hyperglycaemia first detected in pregnancy at 3-6 years post-partum in an urban South African setting
Source: PLoS One. 2022 Feb 9;17(2):e0263529. doi: 10.1371/journal.pone.0263529 (PMC8827431; doi:10.1371/journal.pone.0263529)
Supplement: S1 Table — (DOCX) [file pone.0263529.s001.docx]

S1 Table. Definitions of maternal and neonatal variables and outcomes

| Maternal variables | |
| --- | --- |
| Risk factors for HFDP | Persistent glycosuria, family history of diabetes(first degree), previous unexplained perinatal losses, previous HFDP, history of a macrosomic baby. |
| Pregnancy losses | Includes miscarriage, intrauterine death, ectopic pregnancy and termination of pregnancy and neonatal death |
| Maternal overweight | BMI ≥25 kg/m^2^ |
| Maternal obesity | BMI ≥30 kg/m^2^ |
| Nephropathy | Creatinine > 71ummol/L and MAC >30 mg/L |
| Anaemia | Haemoglobin levels < 11 g/dl |
| HIV-positive | Based on results from rapid or antibody ELISA tests either at index pregnancy or follow up visit |
| Hypertensive disorders of pregnancy | Categorized as:  Pregnancy-induced hypertension (PIH): hypertension presenting >20 completed weeks  Preeclampsia: hypertension with proteinuria  Eclampsia: hypertension with proteinuria and seizures |
| Any obstetric complication | Miscarriage, PIH, pre-eclampsia, eclampsia, urinary tract infection, polyhydramnios, oligohydramnios, abruptio placentae, maternal death, and/or other. |
| Maternal measurements and outcomes | |
| i) Anthropometrics | |
| All Measurements  Waist circumference  Hip circumference  Waist: hip ratio (WHR)  BMI categories  Fat mass index | Measured and recorded three times using a non-elastic tape and final measure being a mean of the 3 readings to one decimal place  Taken at midpoint between iliac crest and lowest part of rib cage in the mid-axillary line  Taken around widest part of hips  As there are no recommended cut-offs for African populations, WHR was categorised as follows:  Normal <0.849 & Central obesity>0.85  BMI <25kg/m^2^ is normal  BMI>25 and <30kg/m^2^ overweight  BMI>30kg/m^2^ obese  Derived from DXA as estimated fat mass (kg) divided by height (m) squared. |
| ii) Glycaemic measures | |
| Diabetes categories  (WHO 2006 criteria) | Diabetes FPG ≥ 7.0mmol/L or OGTT 2hr plasma glucose(PG) ≥11.1mmol/L or HbA1c ≥6.5%  Impaired fasting glucose(IFG) : FPG ≥ 6.1-6.9 and OGTT 2 hour PG < 7.8mmol/L  Impaired glucose tolerance(IGT) FPG < 7.0 and OGTT 2 hour PG ≥7.8 and < 11.1mmol/L |
| Dysglycaemia | Fasting blood glucose level ≥ 5.6mmol/L |
| Insulin resistance | Assessed using the homeostatic model and calculated as fasting glucose (mmol/L) fasting insulin (mU/l)/22.5  HOMA score>1.95 defined as insulin resistance |
| iii) Cardiovascular parameters | |
| Blood pressure measurement | 3 measurements were taken using Omron M6(Omron Kyoto, Japan) on right arm in a sitting position 1 minute apart after 5 minutes at rest, Average of the last 2 readings were recorded |
| Hypertension | Blood pressure of > 140/90 mmHg for at least two measurements |
| Carotid intima media thickness(cIMT) | Thickened cIMT defined as ≥0.8mm |
| Atherosclerotic plaque | Focal structure that extends at least 0.5 mm into the arterial lumen and/or measures 50% or more of the adjacent IMT value and/or has an IMT value greater than 1.5 mm |
| Cardiovascular disease | Self-reported history of coronary artery disease, stroke and or peripheral arterial disease |
| Dyslipidaemia  (local-based criteria) | Dyslipidaemia was defined as either triglyceride ≥ 1.7 mmol/L and/or high-density lipoprotein cholesterol ≤ 1.30 mmol/L and/or LDL ≥ 3.0mmol/L |
| Neonatal outcomes | |
| Prematurity | GA <37 completed weeks |
| Low birth weight | Birth weight <2500 grams |
| Macrosomia | Birth weight ≥ 4000 grams |
