# Supplementary material for: Cardiometabolic outcomes of women exposed to hyperglycaemia first detected in pregnancy at 3-6 years post-partum in an urban South African setting
Source: PLoS One. 2022 Feb 9;17(2):e0263529. doi: 10.1371/journal.pone.0263529 (PMC8827431; doi:10.1371/journal.pone.0263529)
Supplement: S1 Appendix — (PDF) [file pone.0263529.s002.pdf]

Maternal Questionnaire

Study Number ______________________

Maternal Questionnaire

Date of Visit ______________________

Maternal Questionnaire

Data Collector ______________________

Maternal demographic information

Age (Years) ______________________

What is your marital status?
- ◯ Single
- ◯ Married
- ◯ Divorced
- ◯ Other

Please describe ______________________

Ethnic group
- ◯ Black
- ◯ Colored
- ◯ Indian
- ◯ White
- ◯ Other

Please describe ______________________

How many times have you been pregnant, regardless of the outcome? ______________________ (Gravidity)

How many of your pregnancies have successfully gone beyond 20 weeks? ______________________ (Parity)

How many pregnancies have you had following the index pregnancy? ______________________

Pregnancy losses?
- ◯ Yes
- ◯ No

Type of loss	☐ miscarriage<br>☐ stillbirth<br>☐ ectopic<br>☐ termination<br>☐ neonatal death<br>☐ infant/childhood death
Any twin/multiple pregnancies?	◯ Yes<br>◯ No
Any pre-term deliveries? (< 37 completed weeks)	◯ Yes<br>◯ No
If preterm delivery, how many?	______________________
Were you told you had high blood pressure during your pregnancy?	◯ Yes<br>◯ No<br>(Not known with chronic hypertension)
If yes to above, how many times?	______________________
Are you the primary caregiver for your child?	◯ Yes<br>◯ No<br>(Concerning the child you brought to the data collection appointment today.)
Are you the biological mother of this child?	◯ Yes<br>◯ No
What number child is the child you brought in today?	______________________<br>(eg., if they are the oldest child, this would be 1; if born 3rd this would be 3; regardless of any child losses.)

Maternal Education

Have you had an education?	◯ No<br>◯ Yes<br>◯ Don't know
What is your highest level of education attained?	◯ Pre-school<br>◯ Primary school<br>◯ Secondary school<br>◯ Professional or technical training<br>◯ University<br>◯ Don't know

Anthropometry Mother

Study Number ______________________________

MEASUREMENTS

Date of Visit ______________________________

Data Collector ______________________________

Date of Birth ______________________________

1.1) Weight(kg) ______________________________

1.2) Weight(kg) ______________________________

1.3) Weight(kg) ______________________________

Average weight ______________________________

2.1) Height (cm) ______________________________

2.2) Height (cm) ______________________________

2.3) Height (cm) ______________________________

Average height ______________________________

3) BMI ______________________________

4.1.1) Waist circumference (cm) ______________________________

4.1.2) Waist circumference (cm) ______________________________

4.1.3) Waist circumference (cm) ______________________________

Average waist circumference ______________________________

4.2.1) Hip circumference (cm) __________________________________

4.2.2) Hip circumference (cm) __________________________________

4.2.3) Hip circumference (cm) __________________________________

Average hip circumference __________________________________

Waist: Hip ratio __________________________________

Blood Pressure Mother

Study Number

____

4) Blood pressure

Right arm readings 1

Time captured

____

5.4.1) Systolic Right arm 1

____
(mmHg)

5.4.2) Diastolic Right arm 1

____
(mmHg)

5.4.3) Heart rate Right arm 1

____
(bpm)

Right arm readings 2

Time captured

____

5.5.1) Systolic Right arm 2

____
(mmHg)

5.5.2) Diastolic Right arm 2

____
(mmHg)

5.5.3) Heart rate Right arm 2

____
(bpm)

Right arm readings 3

Time captured

____

5.6.1) Systolic Right arm 3

____
(mmHg)

5.6.2) Diastolic Right arm 3

__________________________ (mmHg)

5.6.3) Heart rate Right arm 3

__________________________ (bpm)

Blood Pressure Readings Calculations

Systolic right arm average

____

Diastolic right arm average

____

Heart Rate average

____

High systolic reading! Please report to the nurse

High diastolic reading! Please report to the nurse

Socioeconomic Questionnaire Maternal

Study Number ______________________________

Socioeconomic Status

Household composition

Total number of people living in your home ______________________________

Number of children less than 5 years old living in your home ______________________________

Total number of sleeping rooms in your home ______________________________

How would you describe the home you are living in?

- ◯ House of brick/ concrete block structure on a separate stand or yard or on a farm
- ◯ Traditional dwelling / hut / structure made of traditional materials
- ◯ Flat or apartment in a block of flats
- ◯ Cluster house in complex
- ◯ Townhouse (semi-detached house in complex)
- ◯ Semi-detached house
- ◯ House/flat / room on your homestead
- ◯ Informal dwelling / shack in back yard
- ◯ Informal dwelling / shack not in back yard
- ◯ Caravan / tent
- ◯ Other

Specify ______________________________

What is the main source of drinking water for members of your household?
(Please tick the box that best applies)

- ◯ Piped water (tap) in dwelling
- ◯ Piped water (tap) in site / yard
- ◯ Bottled water
- ◯ Water carrier/ tanker
- ◯ Rain water tank
- ◯ Borehole / well / spring
- ◯ Dam / river / stream
- ◯ Public / communal tap
- ◯ Other

Specify ______________________________

What is this household's main source of water for household use (other than for drinking)?	◯ Regional / local water scheme (operated by municipality or other services provider)<br>◯ Borehole<br>◯ Spring<br>◯ Rain-water tank<br>◯ Dam / pool/ stagnant water<br>◯ River / stream<br>◯ Water vendor<br>◯ Water tanker<br>◯ Other
Specify	__________________________________
What kind of toilet facilities does your household have?	◯ Flush toilet (connected to sewerage system)<br>◯ Flush toilet (with septic tank)<br>◯ Chemical toilet<br>◯ Pit toilet with ventilation (VIP)<br>◯ Pit toilet without ventilation<br>◯ Bucket toilet<br>◯ Other<br>◯ None
Specify	__________________________________
Do you share this toilet with other households?	◯ Yes<br>◯ No

Which of the following do you have in your current home (place where you spent most nights in the past three months)? It does not matter who owns/pays for these things. (Answer all questions)

	Yes	No
Electricity	◯	◯
Fridge	◯	◯
Stove	◯	◯
Vacuum cleaner	◯	◯
Washing machine	◯	◯
MNet/DSTV/Satellite	◯	◯
DVD Player	◯	◯
Motorcar	◯	◯
Television	◯	◯
Telephone (landline)	◯	◯
Cell phone	◯	◯
Computer/Laptop/ Tablet	◯	◯
Internet access	◯	◯

Main fuel for cooking in house	◯ Gas<br>◯ Oil<br>◯ Kerosene<br>◯ Electricity<br>◯ Charcoal<br>◯ Wood<br>◯ Straw or grass<br>◯ Animal dung<br>◯ None

Maternal And Child Medical Questionnaire

Study Number ______________________

Current maternal morbidities

Have you been diagnosed with HIV?
◯ Yes
◯ No

Year of diagnosis ______________________
(Year)

Time of diagnosis in relation to index pregnancy
◯ Before index pregnancy
◯ During index pregnancy
◯ After index pregnancy
(index pregnancy refers to the time you were pregnant with the child you brought in today.)

Which ARV was used during pregnancy? ______________________

If HIV Positive, do you know your CD4?
◯ Yes
◯ No

CD4 value ______________________

Do you know your viral load?
◯ Yes
◯ No

Viral load value ______________________

Diabetes status

Have you been diagnosed with diabetes mellitus following the index pregnancy?
◯ No
◯ Yes
◯ Don't know

Year of diagnosis ______________________
(year)

Which subtype?
◯ Type 2
◯ Type 1

What treatment are you currently on?
◯ Oral: Metformin or Glibenclamide or both
◯ Insulin
◯ Both orals and insulin
◯ Other
◯ Unknown

Which type of oral agent are you using? __________________________________

Please describe __________________________________

Did you remain on treatment for diabetes after delivery for the index pregnancy?
- ◯ No
- ◯ Yes
- ◯ Don't know

( )

If yes to above, what treatment were you on?
- ◯ Oral: Metformin or Glibenclamide or both
- ◯ Insulin
- ◯ Both orals and insulin
- ◯ Unknown

Did you attend the postpartum visit following your index pregnancy for an oral glucose tolerance test (OGTT)?
- ◯ No
- ◯ Yes
- ◯ Don't know

What was the result?
- ◯ Diabetes
- ◯ No diabetes
- ◯ Unknown

Please describe __________________________________

What was your main reason for not attending?
- ☐ Not told or aware of appointment
- ☐ Too busy or no time to attend
- ☐ Did not know I had to come if I had no symptoms
- ☐ I was not told importance of the visit
- ☐ I had no transportation to the clinic
- ☐ Other

Overall, how many GDM pregnancies have you had (including the index pregnancy)? __________________________________

Have you been diagnosed with any of these conditions?

	No	Yes	Don't know
Have you been diagnosed with hypertension?	◯	◯	◯
Have you been diagnosed with cholestrol problems?	◯	◯	◯
Any other chronic medical illnesses?	◯	◯	◯

Are you taking lipid lowering agents?
- ◯ Yes
- ◯ No

If yes, please specify __________________________________

Mother -CVD event

	No	Yes	Don't know
Heart attack	◯	◯	◯
Stroke	◯	◯	◯
Peripheral vascular disease-gangrene/amputation of limb	◯	◯	◯

Was it before or after index pregnancy?
◯ before index pregnancy
◯ after index pregnancy

Maternal and family lifestyle factors

Did you partake in any recreational (not work related) exercise following the index pregnancy?
◯ Yes
◯ No

If yes to above, how many times a week?
◯ 1 (once)
◯ 2 (twice)
◯ 3 (thrice)
◯ 4 (4 times)
◯ 5 (5 times)
◯ more than 5 times
◯ Don't know

How many years?
◯ < 1 year
◯ 1-2 years
◯ 3-5 years
◯ >5 years

Do you consume any alcohol?
◯ Yes
◯ No

If yes to previous, how many times in a week? ______________________

If yes, how many units at a time? ______________________

Did you drink alcohol during your pregnancy with this child?
◯ No
◯ Yes
◯ Don't know

How many times in a week? ______________________

How many glasses at a time? ______________________

Do you smoke cigarettes
◯ Yes
◯ No

If yes to previous, how many cigarettes per day? ______________________

How many years?	__________________________________
Did you smoke cigarettes during your pregnancy with this child?	◯ No<br>◯ Yes<br>◯ Don't know
How many cigarettes per day?	__________________________________
Any family history (1st/2nd degree relatives) with a cardiovascular event/s? (heart attack or stroke)	◯ No<br>◯ Yes<br>◯ unknown
Do you have any family history of diabetes?	◯ No<br>◯ Yes<br>◯ Don't know
Do your mother, father, or any siblings have diabetes?	◯ 1) Yes<br>◯ 2) No<br>◯ 3) Don't know<br>(2nd degree relative)
Do your grandparents, aunts, uncles, nephews or nieces, have diabetes?	◯ 1) Yes<br>◯ 2) No<br>◯ 3) Don't know<br>(2nd degree relative)
Does your child's biological father have diabetes?	◯ 1) Yes<br>◯ 2) No<br>◯ 3) Don't know

Early nutrition

Did you breastfeed following this pregnancy?	◯ No<br>◯ Yes<br>◯ Don't know
How long did you breastfeed for?	__________________________________<br>(months)
Did you breastfeed exclusively following this pregnancy?	◯ No<br>◯ Yes<br>◯ Don't know
How long did you breastfeed exclusively for?	__________________________________<br>(months)
Was you child hospitalized in the first two years of life?	◯ No<br>◯ Yes<br>◯ Don't know
How often was the child hospitalised in the first two years of life?	__________________________________

What was the reason for hospitalization?

____

Attendance of pre-primary

Does your child attend pre-school, pre-primary, creche, or a similar facility?

- ◯ No
- ◯ Yes
- ◯ Don't know

How many hours per week does your child spend there?

____
(Hours)

At what age did your child first attend the facility?

____
(Months)

Contraception

Have you been on any pregnancy prevention (contraception) since the index pregnancy?

- ◯ No
- ◯ Yes
- ◯ Don't know

Which form of contraception?

- ☐ Oral (pill)
- ☐ Injectable
- ☐ Other

If other, which?

____

If oral, progesterone only oral contraception?

- ◯ Yes
- ◯ No
- ◯ Unknown

Questionnaire completed?

- ◯ Yes
- ◯ No

If no to previous, please provide explanation for incompleteness.

____
