# Supplementary material for: Correction: Cardiometabolic outcomes of women exposed to hyperglycaemia first detected in pregnancy at 3–6 years post-partum in an urban South African setting
Source: PLoS One. 2023 Aug 8;18(8):e0289985. doi: 10.1371/journal.pone.0289985 (PMC10409261; doi:10.1371/journal.pone.0289985)
Supplement: S1 File — (PDF) [file pone.0289985.s001.pdf]

PLOS ONE

RESEARCH ARTICLE

Cardiometabolic outcomes of women exposed to hyperglycaemia first detected in pregnancy at 3-6 years post-partum in an urban South African setting

Veronique Nicolaou[1,2]*, **Larske Soepnel**[1,3], **Kenneth Huddle**[2], **Kerstin Klipstein-Grobusch**[3,4], **Naomi S. Levitt**[5], **Shane A. Norris**[1,6]

1 Faculty of Health Sciences, SAMRC/Wits Developmental Pathways for Health Research Unit, Department of Paediatrics, University of the Witwatersrand, Johannesburg, South Africa, **2** Faculty of Health Sciences, Department of Internal Medicine, Chris Hani Baragwanath Academic Hospital, University of Witwatersrand, Johannesburg, South Africa, **3** Julius Global Health, Julius Center for Health Sciences and Primary Care, University Medical Center Utrecht, Utrecht University, Utrecht, The Netherlands, **4** Faculty of Health Sciences, Division of Epidemiology and Biostatistics, School of Public Health, University of the Witwatersrand, Johannesburg, South Africa, **5** Faculty of Health Sciences, Chronic Disease Initiative for Africa, Department of Medicine, University of Cape Town, Cape Town, South Africa, **6** Global Health Research Institute, School of Human Development and Health, University of Southampton, Southampton, United Kingdom

* docvic9@gmail.com

Abstract

Background

Hyperglycaemia first detected during pregnancy(HFDP) has far-reaching maternal consequences beyond the pregnancy. Our study evaluated the cardiometabolic outcomes in women with prior HFDP versus women without HFDP 3–6 years post-partum in urban South Africa.

Design and methods

A prospective cohort study was performed of 103 black African women with prior HFDP and 101 without HFDP, 3–6 years post-partum at Chris Hani Baragwanath Academic Hospital, Soweto. Index pregnancy data was obtained from medical records. Post-partum, participants were re-evaluated for anthropometric measurements, body composition utilizing dual energy X-ray absorptiometry(DXA) and biochemical analysis (two-hour 75gm OGTT fasting insulin, lipids, creatinine levels and glucose levels). Cardiovascular risk was assessed by Framingham risk score(FRS). Carotid intima media thickness(cIMT) was used as a surrogate marker for subclinical atherosclerosis. Factors associated with progression to cardiometabolic outcomes were assessed using multivariable logistic and linear regression models.

Results

Forty-six(45.1%) HFDP women progressed to diabetes compared to 5(4.9%) in non HFDP group(p<0.001); only 20(43.4%) were aware of their diabetic status in the whole group. The

OPEN ACCESS

Citation: Nicolaou V, Soepnel L, Huddle K, Klipstein-Grobusch K, Levitt NS, Norris SA (2022) Cardiometabolic outcomes of women exposed to hyperglycaemia first detected in pregnancy at 3-6 years post-partum in an urban South African setting. PLoS ONE 17(2): e0263529. https://doi.org/10.1371/journal.pone.0263529

Editor: Guoying Wang, Johns Hopkins University Bloomberg School of Public Health, UNITED STATES

Received: August 18, 2021

Accepted: January 20, 2022

Published: February 9, 2022

Copyright: © 2022 Nicolaou et al. This is an open access article distributed under the terms of the Creative Commons Attribution License, which permits unrestricted use, distribution, and reproduction in any medium, provided the original author and source are credited.

Data Availability Statement: ***PA AT ACCEPT: Please follow up with authors for data available at accept*** Dataset is available from the figshare repository (accession number 10.6084/m9.figshare.17004064).

Funding: The author(s) received no specific funding for this work.

Competing interests: No. The authors have declared that no competing interests exist.

odds(OR, 95% confidence interval(CI)) of progressing to type 2 diabetes(T2DM) and metabolic syndrome(MetS) after correcting for confounders in the HFDP group was 10.5(95% CI 3.7–29.5) and 6.3(95%CI 2.2–18.1), respectively. All visceral fat indices were found to be significantly higher in the HFDP group after adjusting for baseline body mass index. Ten-year estimated cardiovascular risk(FRS) and mean cIMT was statistically higher in the HFDP group(8.46 IQR 4.9–14.4; 0.48 mm IQR 0.44–0.53 respectively) compared to the non-HFDP group(3.48 IQR 2.1–5.7; 0.46mm IQR 0.42–0.50) respectively and this remained significant for FRS but was attenuated for cIMT after correcting for confounders. HIV did not play a role in progression to any of these outcomes.

Conclusion

Women with a history of HFDP have a higher risk of cardiometabolic conditions within 6 years post-partum in an urban sub-Saharan African setting.

Introduction

The non-communicable diseases(NCD) burden remains the leading cause of death worldwide, with diabetes and cardiovascular diseases(CVD) accounting for almost half the burden. In South Africa, diabetes and CVD are the second and third leading cause of death since 2014. In high-income countries, hyperglycaemia first detected during pregnancy(HFDP) has been associated with a sevenfold higher overall incidence of type 2 diabetes mellitus(T2DM) within the first decade following delivery [1] and an increased risk of CVD [2] and the metabolic syndrome(MetS) [3].

Prevalence figures for HFDP in Africa remain limited, with a few studies in South Africa demonstrating that 9–25% of women have HFDP [4–6]. These figures may underrepresent the disease burden since risk-factor based screening and varying diagnostic tests and strategies are currently employed. Whilst numerous studies have explored the short term maternal and neonatal outcomes following HFDP, long-term outcomes for the African continent remain limited to a mere three studies. One study performed amongst a predominantly mixed ethnic ancestral group in Cape Town, South Africa demonstrated a 48% progression rate to T2DM at 5–6 years following the index pregnancy [7], although no control group was included. A high CVD risk and prevalence of MetS(60.9%) was demonstrated in the same cohort [8]. A further smaller study amongst the same ethnic group from the same region established that the prevalence of T2DM at six-weeks post-partum was 27% [9].

Though racial and ethnic disparities for progression to T2DM following HFDP is well known with African American women being particularly vulnerable [10] owing to acculturation, lifestyle factors arising from social determinants in addition to genetic susceptibility and traditional risk factors [11], to our knowledge, the long-term CVD and metabolic impact of HFDP has not previously been explored in Black African women or compared to women in this setting without a history of HFDP.

Obesity, a well-known risk factor of HFDP, T2DM and CVD, is commonly encountered amongst black South African women(40.9%) and accounts for an estimated 87% of their diabetic risk [12,13]. Obesity and weight-related characteristics including pre-pregnancy body mass index (BMI), post-pregnancy BMI and weight gain following the pregnancy, all have been shown to increase the risk of progression to T2DM following HFDP [14]. The

pathophysiological mechanism behind this lies in the differential impact of regional fat deposition, with upper body(android and visceral fat) and lower body fat (gynoid and leg fat) showing directionally opposite associations with these risks [15] which too has been appreciated in black SA women [16,17]. However, the impact of body composition characteristics on progression to T2DM, MetS and CVD following an HFDP pregnancy has yet to be explored in this setting.

Moreover, little is known about the impact of human immunodeficiency virus (HIV), which the healthcare sector in Africa is faced with, in addition to the growing burden of NCDs, on CVD and metabolic risk in the context of HFDP. The additional metabolic risk attributable to HIV is debatable, with an overall prevalence of glucose metabolism disorders in HIV-infected individuals on antiretroviral therapy(ART) in Africa ranging from 3–33.5% [18]. A meta-analysis of 5 case-control studies in Africa did not demonstrate a significant relationship between HIV and exposure to ART and the prevalence of T2DM, as encountered in other studies in Europe and North America.

Given the growing prevalence of HFDP in South Africa and its well-known role in the intergenerational transmission of NCD, we sought to explore its impact on maternal CVD risk, development of T2DM and MetS in a group of Black African women with and without a prior history of HFDP. Secondary aims of our study were to explore how body composition differs between the two groups and if HIV-infection impacted the development of these outcomes following pregnancies with and without HFDP. To our knowledge this is the first study of its kind in Africa.

Study design and population

The study setting was the Chris Hani Baragwanath Academic Hospital(CHBAH), located in urban Soweto, South Africa. Between March and November 2019, we conducted a prospective cohort study in women previously diagnosed with HFDP (HFDP group) and women who tested negative for HFDP (non-HFDP group) using the same diagnostic test and criteria between February 2014 to January 2017. Both groups of women were derived from the same specialist clinic though were identified differently. The HFDP group were selected first and consisted mostly of women who were identified by risk-factor based screening and had attended a specialised gestational endocrine clinic at CHBAH for HFDP with their pregnancy characteristics and outcomes have been previously published [19]. A subgroup of the HFDP women were referred to the specialist clinic as a result of universal screening being performed by a research study [5]. The "control group", were women who tested negative for HFDP for the same time period and had undergone universal screening as part of a previous study [5]. Women were diagnosed using a 75-gram 2-hour oral glucose tolerance test(OGTT) with International Association of Diabetes in Pregnancy Study Group(IADPSG) criteria. HFDP comprised of "true" gestational diabetes mellitus (GDM) and "overt" diabetes in pregnancy (DIP).

Participants were recruited telephonically and if unsuccessful traced by visiting their home address. Of the initial 319 HFDP cases identified, 206 were non-contactable/traceable, 4 declined to participate, 6 were pregnant. Difficulties tracing participants following delivery was mostly due to relocation or change of contact details. There were 845 women who screened negative for HFDP identified from the database of the previous study of which 103 women were contacted in a random order until the number of mothers were the same as those in the HFDP group. Two hundred and four participants were enrolled at follow up, of which 103 had confirmed HFDP and 101 did not.

Sample size calculation

Sample size calculations were calculated for each of the three main outcomes(T2DM, MetS and CVD risk) using a two-sample proportion test based on population parameters using a 5%

margin of error, a confidence interval of 95% and power of 80% to detect the effect. Given the reported estimated risk of developing T2DM following a HFDP pregnancy of 20–60% and 12% [1,20] in the background population a sample size of 15 per group was calculated. The minimum total sample size calculated for the outcome MetS was 64 given the risk of 40% following HFDP [3] and background prevalence of MetS of 10%. A size of 98 per group was needed based on a reported estimated risk of developing CVD of 17.6% [2] following HFDP vs. 7% in the background population. The sample size needed to establish CVD risk informed our final sample size.

Data collection

a) Questionnaire. A self-reported questionnaire was captured at the follow-up visit and incorporated maternal demographics, marital status, various socioeconomic parameters (SES), obstetrical history, maternal complications and outcomes and postnatal factors (history of CVD risk factors, any vascular event/s, risk factors for the development of T2DM including recurrent HFDP pregnancies, breastfeeding following the index pregnancy, family history of diabetes or the presence of diabetes). Use of cholesterol-lowering or antihypertensive medication, smoking status and pack year history and ethanol consumption(ml/day) based on the quantity and frequency and physical activity was captured. Contraceptive use was self-reported and was categorized as none, oral contraceptives or injectable contraceptives and type. HIV status and therapy were noted where applicable. (See S1 Table for relevant definitions of maternal variables noted). The questionnaire (S1 Appendix)) was informed by the literature and adapted from several existing standard and recognised sources [21] in order to incorporate relevant factors.

b) Anthropometrics. Subjects underwent a physical examination for weight, height, waist and hip circumference and blood pressure measurements utilising standardised methods by qualified trained research assistants. Height (cm) was recorded to one decimal place using a wall mounted Holtain stadiometer(Crymych, UK) with subjects standing on a flat surface at a right angle to the vertical board of the stadiometer. Weight(kg) was measured on a SECA digital scale (Hamburg, Germany), to the nearest 0.1kg, which was calibrated and standardised using a weight of known mass. Participants wore light clothing and were asked to remove their shoes and socks. Blood pressure(mmHg) measurements were taken. Details pertaining to each measurement is outlined in S1 Table.

Dual–energy x–ray absorptiometry (DXA)(Hologic Discovery-A (S/N83145), Bedford, MA, USA) was used to determine whole-body composition since BMI alone is not an accurate indicator of body composition. This included subtotal(whole-body minus head) fat mass and fat-free soft tissue mass. Regional body fat, namely trunk, arm, leg, android, and gynoid fat mass(expressed in kg and as a percentage of subtotal fat mass, (% FM)) were measured using DXA cut-off lines positioned at standard anatomical positions, as defined in the software (software version apex 4.2.0). FMI (kg/m^2) was calculated using height and body fat mass which offers superiority over BMI as a marker of obesity since the index is based on fat mass, not body weight, which is a combination of fat and lean components. In addition, abdominal visceral adipose tissue(VAT) and subcutaneous adipose tissue(SAT) were estimated using algorithms included in the DXA software, which have been shown to perform as well as clinical computed tomography [22]. During data collection, a phantom scan was performed each morning to) determine the coefficient of variance of the DXA machine and the coefficient of variance (CV) was less than 0,5% for all parameters. CVs for DXA parameters were <2% for total fat mass, and 1% for fat–free soft tissue mass(FFSM).

c) Biological samples and OGTT. Point of care testing was performed for haemoglobin (Hemocue^r Hb 201) and HIV(Homemed HIV1/2 rapid test kit). An early morning midstream

urine sample was collected from each participant to exclude pregnancy and to perform bedside testing with a urine dipstick(Roche Combur) screening for glycosuria, albumin/protein, evidence of infection or renal disease.

A finger prick(OneTouch) fasting capillary glucose was performed at baseline and the OGTT was commenced irrespective of the result. At baseline, blood samples were drawn by a trained nurse after an 8-hour overnight fast for measurement of serum creatinine, lipogram, Hba1c, fasting insulin and glucose. This was followed by ingestion of 75g glucose in 250ml water, Blood samples for glucose were drawn at baseline and 2 hours. Those with self-reported diabetes diagnosis, which was confirmed by either medical card record or drugs in-use only had fasting bloods drawn. Specimens were centrifuged and stored at -80˚C within 30 min of being drawn. Categories of glucose intolerance were defined applying the 2006 WHO criteria [23]. (S1 Table).

d) Carotid and femoral imaging. Ultrasonographic assessment of the common carotid artery(CCA) and femoral artery(CFA) was performed(Linear-Array 12L-RS transducer with a B-mode Logic E Ultrasound machine, GE healthcare, CT, USA), to assess for intima media thickness(IMT) and for the presence of plaque. The IMT measurement was then performed on the posterior wall of the common carotid artery and common femoral artery in an area free of plaque, defined as the distance between two echogenic lines represented by the lumen-intima interface and media-adventitia interface of the arterial wall. The ultrasound machine software then detected the intima-lumen and the media-adventitia interfaces and calculated the minimum, maximum, and mean common carotid IMT(cIMT) and femoral IMT in millimetres and to 2 decimal places [24,25]. All patients were positioned supine with the neck slightly hyperextended and rotated in the opposite direction to the probe. A 45-degree angle wedge pillow was used to standardize lateral rotation. Measurements were performed by one observer with intra-observer variability 1.1%.

e) Biochemistry and lab analyses. Plasma glucose was measured using Randox Rx Daytona chemistry analyser using enzymatic methods(Randox Laboratory Ltd, London, UK) glycosylated haemoglobin a1c(HbA1c) was measured using the Bio-Rad D-10™ Haemoglobin analyser using the HPLC method (catalogue number, 2200101) (Bio-Rad Laboratories, Inc. CA, USA). The precision and trueness of the Randox Rx Daytona chemistry analyser were verified using the clinical and laboratory standards institute document EP15. Coefficients of variation calculated from running 30 separate samples at 3 different times were 0.7% for glucose and 1.8% for HbA1c. Lipids including HDL, low-density lipoprotein(LDL), triglycerides(TG), total cholesterol concentrations were analysed on the Randox Rx Daytona chemistry analyser using enzymatic colorimetric (catalogue number, CH8311(HDL), CH8312(LDL), TR8332(trigs), CH8310(Chol)) Randox Laboratories Ltd., London, UK). Enzymatic colorimetric assays were used to measure TG, total cholesterol and HDL cholesterol using the Roche modular auto analyser, while low-density lipoprotein cholesterol was calculated using direct methods/Friedewald formula. Fasting serum insulin concentrations were measured on the Immulite® 1000 Immunoassay system using the chemiluminescent method(catalogue number lkin1/ catalogue number, 10381429) (Siemens) chemiluminescent healthcare GMBH, Henkestr, Germany). Serum creatinine concentrations were analysed on the Randox Rx Daytona chemistry analyser using enzymatic methods (catalogue number, CR8317) (Randox Laboratories Ltd., London, UK). CVs calculated from running 40 separate samples in duplicate were, 0.8% for HDL and total cholesterol, 1.19% for TG and 3.9% for insulin and 0.7% creatinine.

Outcomes

The primary outcome was progression to and time to developing T2DM between women with HFDP(sub-categorised; GDM and DIP) and women without HFDP following their index

pregnancy. Secondary outcomes included comparison of body composition measures, progression to MetS and CVD risk(utilising two surrogate measure, Framingham Risk Score (FRS) and cIMT between the groups. MetS was defined using the harmonised criteria [26]. Gender specific prediction for 10-year CVD risk was calculated using the modified Framingham risk score 2008 (FRS) [27] (See S1 Table for definitions).

Ethics

This study was approved by the Human Research Ethics Committee at the University of the Witwatersrand(M180316). Informed consent, both verbal and written, was obtained from participants prior to enrolment in the study.

Statistical analysis

Data was captured using REDCap [28] (Vanderbilt University, Nashville, USA) and analysed using Stata software 13.0(College station, USA) [29]. Sensitivity analysis to address potential bias of missing data was performed. Mean and standard deviations were reported for normally distributed continuous variables(anthropometric parameters) and medians and interquartile ranges for non-normally distributed measured data (all other continuous variables). Number and percentages for categorical variables(chronic hypertension, family history of diabetes etc). Statistical differences between three groups(control, GDM and DIP), were tested using Analysis of Variance(ANOVA) or Kruskal-Wallis test. For categorical variables, Chi-squared test and Fischer's exact(small frequencies) was utilised. The statistical significance level was set at two-sided p-value <0.05.

Crude odds ratio(OR 95% CI) and multivariable adjusted odds ratio(aOR 95% CI) for T2DM, MetS, and 10-year cardiovascular risk calculated using FRS were estimated from logistic regression models. Covariates evaluated as potential confounders based on a priori hypotheses are included in Table 3. Covariates were excluded as confounders if they were not associated with both the dependent and independent variable (exposure to HFDP), $p<0.05$.

Further multivariable models were designed to explore the relationship of maternal factors associated with the relevant outcomes adopting a chronological approach in which maternal factors present either at pre-pregnancy(distal model 1), index pregnancy(intermediate model 2) and post-partum(proximal model 3) were assessed using logistic or linear regressions. The final model combined all variables from the three models. The outcomes explored in these multivariate models were fat mass index FMI(continuous, as surrogate for adiposity), T2DM (binary) and cIMT(continuous). The independent variables were identified using univariate analysis for each outcome. HFDP, an independent variable for all models, was categorised according to degree of dysglycaemia as GDM and DIP. Both BMI and WHR were used as continuous variables for the purposes of the models. For logistic regression model diagnostics, results are expressed as OR and 95% confidence intervals(CI) and we assessed the following: linearity assumption using the Lowes graph, multicollinearity using variance inflation factors, model specification using the C-statistic, and confirmed the fit of the model using the Hosmer-Lemeshow goodness-of-fit test. We also checked for outliers. For linear regressions cIMT was log-transformed to increase normality of residuals. Results are expressed as beta coefficient and 95% CI. In order to assess the influence of HIV on the outcomes, it was included as binary variable into each of the multivariate models exploring maternal factors and outcomes.

Results

There were 103 women recruited in the HFDP group and 101 in the non-HFDP group after all exclusions were applied. There was <1% missing data and no biases detected through the sensitivity analyses. Details are shown in the study flow diagram (Fig 1).

a) Baseline and follow up demographics, maternal factors, anthropometrics, and biochemical parameters between the groups

The majority of the cohort was of Black African ancestry (n = 198, 97%). Relevant baseline characteristics during the index pregnancy and at follow-up for the HFDP group and non-HFDP groups are shown in Table 1. Of the participants with prior HFDP, 45(43.7%) had "overt" diabetes in pregnancy (DIP) with the remaining 58(56.3%) classified as "true" GDM (GDM). When comparing the HFDP vs. non-HFDP groups at first booking during their index pregnancy, the median age was higher for the HFDP group, 32.5(29–38 IQR) vs. 29.5 (25–34 IQR) respectively, with a median follow-up period of 3 years (IQR3-4) and their median BMI were 35.2(IQR30.6–39.8) and 29.5(IQR 25.0–32.9). Baseline prevalence of HIV during pregnancy was lower in the HFDP group 13(12.7%) vs. 20(19.8%) amongst the non-HFDP group and this remained the case at follow-up. All women with HIV were on fixed dose combination of treatment whilst the majority(80%) were diagnosed at or before their index pregnancy. Forty-five(43.7%) of the women in the HFDP group experienced an obstetric complication vs.16(15.8%) in the non-HFDP group. When comparing the DIP and GDM groups, the only variables significantly different at baseline were glucose values on OGTT testing and exposure to therapeutic agents, with more in the DIP group being exposed to insulin 22(48.9%) vs. 8 (14.8%).

At follow-up, the HFDP group remained obese (32.8 (29.1–39.2)) with elevated anthropometric measures, higher blood pressure measurements and glucose profiles when compared to the non-HFDP group. Their overall socioeconomic status was lower than those for the non-HFDP group.

b) Stratified analysis of maternal outcomes including diabetes, cardiovascular risk and metabolic syndrome by HFDP subtypes: DIP and GDM (Table 2)

Of the HFDP group, 46(44.6%) progressed to diabetes compared with 5(4.9%) in the non HFDP group(p<0.001). Only 20(42.5%) of the entire group were aware of their diabetes status and the average time to event was 30 months (SD +/-1.32). Both dysglycaemia(57 (55.9%) vs.14 (13.9%)) and insulin resistance (92 (90.2%) vs. 69 (68.3%), p<0.001)) were significantly higher in the HFDP group. Within the HFDP group, all measures of dysglycaemia, insulin resistance and progression to T2DM were higher among the DIP group compared to the GDM group.

All CVD risk factors were higher in the HFDP group including diabetes 46(44.6%), hypertension 27(26.1%), dyslipidemia 85(82.5%), family history of CVD 20(19.4%), history of ever smoking 10 (9.7%) and central obesity 66(64.1%), though smoking and family history of CVD were not significantly different between the groups. Overall, the calculated FRS was significantly higher in the HFDP group 8.46(4.9–14.4) vs. 3.48(2.1–5.7) p<0.001, with an intermediate to high-risk score being present in over 40% of the HFDP individuals 42 (42%) vs. 8 (8%) p<0.001. Within the HFDP subtypes, DIP displayed a significantly higher intermediate-high risk category cardiovascular score of 26 (60.4%) vs. 16 (28%) in the GDM group. The overall median cIMT measurement, though within normal limits in all participants, was significantly

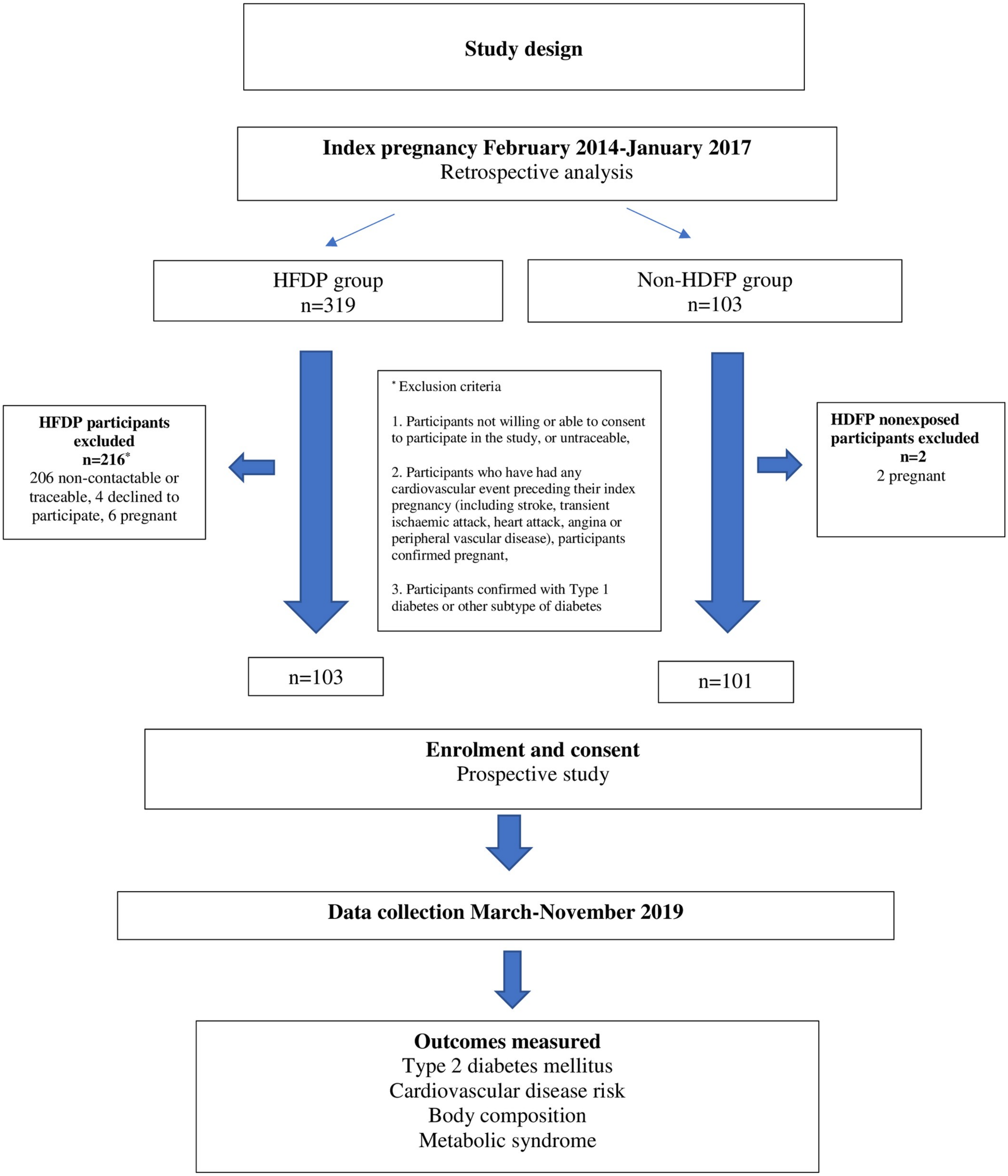

Fig 1. Flow diagram of the study and eligible participants.

https://doi.org/10.1371/journal.pone.0263529.g001

higher in the HFDP group, with a cIMT of 0.48 (IQR 0.44–0.53) vs. 0.46 (IQR 0.42–0.5) p = 0.037, though this was no longer significant after adjusting for age. No atherosclerotic plaque was noted at either carotid or femoral sites in any of the participants and there was only one reported event of CAD and one of cerebrovascular accident. Though these measured outcomes may be attenuated after correcting for differences in maternal age and BMI at index pregnancy, this has been explored in the subsequent regression models (Tables 5–7).

The prevalence of the MetS was higher in the HFDP group, 42(40.8%) vs. non-HFDP group 6(5.9%) p<0. 001.Between the two subtypes of HFDP, more women in the DIP arm had MetS 25(55.6%) vs 17(29.3%).

c) Body composition measures between the groups (Table 3)

Ninety-nine HFDP and 96 of the non HFDP women(n = 195) had a DXA scan performed. All body composition variables, except total percent body fat, were significantly higher in the exposed vs. nonexposed groups, including the fat mass index (FMI) 13.9(11.7–16.3) vs.12.3 (8.9–14.6) p = 0.0008 and VAT:SAT ratio 0.20 vs.0.16. p<0.001. In particular, measures of visceral adipose tissue(VAT) were elevated in HFDP women. VAT volume was 517cm^3(IQR372.6–610.2) vs. 322 cm^3(IQR 219.2–469.2) p <0.001 and percentage trunk fat of 39.8%(IQR 35–43.6) vs 36.5%(IQR 29.4–42.5) p = 0.0066 with android:gynoid ratio at 0.93 (0.86–1.02) vs 0.86(0.75–0.95) p <0.0001.After adjusting for BMI at baseline, only visceral adiposity indices remained significant between the groups.

d) Strength of association of prior HFDP and progression to diabetes, MetS and CVD risk (Table 4)

The crude OR(15.5(CI 5.8–41.3, p<0.001)) for progressing to T2DM in the HFDP group remained significant(adjusted aOR 10.5(CI 3.7–29.5) p<0.001) after correcting for confounders. This significant risk persisted within the subtypes of HFDP, with a greater aOR 27.6(8.7–87.4) in the DIP group. The crude OR for prior HFDP and its association with MetS was 10.9 (4.3–27.1 p<0.001) vs. aOR of 6.3(CI 2.2–18.1), which remained significant (p = 0.004) after adjusting for factors present at the index pregnancy including maternal age, parity, systolic blood pressure(SBP) and BMI. Additionally, the aOR within the HFDP subtypes and MetS risk remained significant. In the HFDP group, the association with an intermediate-high 10-year estimated CVD risk based on FRS was significant for crude OR 8.4(3.6–19.1) p<0.001; and when adjusting for confounders including factors at index pregnancy including age, BMI, SBP(aOR of 4.3(1.6–11.5, p = 0.003) and this significance was retained in the DIP group.

e) Factors associated with FMI and progression to type 2 diabetes and cardiovascular risk using multiple variable logistic and linear regression

i) Factors associated with FMI. Linear regression models of relevant independent maternal factors present at pre-pregnancy(distal), pregnancy(intermediate) and post-partum(proximal) associated with log FMI is shown in Table 5. In the distal model only multiparty was significantly associated with FMI. In the intermediate model, BMI measured at first visit in pregnancy was significantly associated with the outcome, with BMI difference (post-partum BMI- pregnancy BMI) being the only significant factor in the proximal model. In the final model, only initial BMI in pregnancy and BMI difference was significantly associated with the outcome log FMI.

ii) Progression to T2DM (Table 6). Multivariate logistic regression models examining the association between relevant maternal risk factors and progression to diabetes found that

Table 1. Maternal demographics, characteristics and outcomes at index pregnancy and follow-up.

**Index pregnancy**		**HFDP group**			**Non-HFDP group**
		Total group<br>n = 103	GDM<br>n = 58	DIP<br>n = 45	n = 101
Age(years)	(median/IQR†)	32.5 (29–38)	33(29–38)	32(30–37)	29.5 (25–34)
Primigravida	n (%)	8 (7.8)	5(8.8)	3(6.8)	54 (53.4)
GA[1] at presentation (weeks)	(median/IQR†)	n = 102<br>30(26–33)	n = 57<br>30(27–33)	n = 45<br>30(24–32)	n = 101<br>26(25–27)
BMI[2] at first visit	(median/IQR†)	n = 96<br>35.2 (30.6–39.8)	n = 53<br>34.3(30.5–38.2)	n = 43<br>37.2(31.6–40.9)	n = 101<br>29.5(25.0–32.9)
BP[3], (mmHg)<br>Systolic BP<br>DBP BP	(median/IQR†)	n = 98<br>121(112–129)<br>73(64–79)	n = 53<br>124(112–129)<br>71(62–77)	n = 43<br>119(113–129)<br>74(66–79)	n = 101<br>109(102.5–114.5)<br>69(64–73)
HIV[4] positive	n (%)	13(12.7)	11(18.9)	2(4.4)	20(19.8)
OGTT[5] results, mmol/L<br>Fasting plasma glucose<br>60 mins<br>120 mins	(median IQR†)	n = 95<br>6.1(5.3–7.4)<br>10.6(8.6–11.9)<br>10.0(8.0–11.9)	n = 55<br>5.6(5.16–6.1)<br>9.25(8.1–10.65)<br>9.0(7.4–10)	n = 40<br>7.6(6.5–9.3)<br>12.2 (11.4–14.3)<br>12.2 (11.35–15.05)	n = 101<br>4.0(3.6–4.4)<br>5.8 (5.0–6.7)<br>5.2(4.5–6.3)
GA at delivery weeks<br>Delivery <37 weeks	(median/IQR†)<br>n (%)	n = 100<br>38(37–38)<br>24(24)	n = 55<br>38(37–38)<br>12(21.8)	n = 45<br>37(36–38)<br>12(26.7)	n = 90<br>39(37–40)<br>13(14.4)
Obstetric complications<br>Caesarian section<br>HDP[6]	n (%)	45(43.7)<br>67(66.3)<br>12(11.6)	21(36.2)<br>39(68.4)<br>6(10.3)	24(53.3)<br>28(63.6)<br>6(10.3)	16(15.8)<br>59(58.4)<br>11(10.9)
Exposure to OHA[7]<br>Metformin<br>Glibenclamide<br>Exposure to insulin	n (%)	n = 99<br>65(65.7)<br>31(31.3)<br>34(33.0)	n = 54<br>38(70.4)<br>12(22.2)<br>8(14.8)	n = 45<br>27(60)<br>19(42.2)<br>22(48.9)	N/A<br>N/A<br>N/A
Neonatal outcomes<br>Birth weight<br>LBW[8]<br>Macrosomia<br>Anomalies	(median/IQR†)<br>n (%)	n = 99<br>3065 (2665–3430)<br>18(18.1)<br>5(5.0)<br>3(2.9)	n = 56<br>3042.5(2650–3345)<br>11(19.6)<br>3(5.3)<br>2(3.5)	n = 43<br>3140(2765–3685)<br>7(16.3)<br>2(4.6)<br>1(2.2)	n = 100<br>3040(2792.5–3357.5)<br>17(17)<br>5(5)<br>2(2.1)
**Demographic, anthropometric and biochemical status at 3–6 years after the index pregnancy**		**HFDP group**			**Non- HFDP group**
		**Total group<br>n = 103**	**GDM<br>n = 58**	**DIP<br>n = 45**	**n = 101**
Age (years)	(median/IQR†)	37.3 (33.1–42.7)	38.1(32.8–42.7)	37.1(33.9–41.7)	34.0(29.2–37.3)
Primigravida	n (%)	6(5.8)	3(5.2)	3(6.7)	13(12.8)
Time at follow-up (years)	(median/IQR†)	3(3–4)	3(3–4)	3(3–4)	3(3–4)
SES[9]<br>1)Marital status, married<br>2) Education Secondary and higher<br>3) Household items<br>4) Housing density	n(%)<br>(mean SD ‡)<br>(median/IQR†)	38(36.9)<br>66(65.3)<br>7.9(2.1)<br>2.5(2–3)	20 (34.4)<br>40(71.4)<br>7.8(2.0)<br>2.5(2–4)	18(40)<br>26(57.8)<br>7.9(2.3)<br>2.5(2–3)	15(14.8)<br>73(23)<br>8.3(1.9)<br>2.0(1.5–3)

(*Continued*)

Table 1. (Continued)

BP[3], (mmHg)<br>Systolic BP<br>Diastolic BP	(median/IQR[†])	115.1(108–126.6)<br>82.6(76.6–90.6)	115(106.6–125)<br>81(73.3–89.6)	117.5(112.-131.8)<br>84.8(77.6–90.6)	113.7(104.6–120.3)<br>79(73.3–86.6)
HIV positive	n(%)	n = 102<br>19(18.6)	n = 57<br>14(24.1)	n = 45<br>5(11.1)	n = 101<br>25(24.7)
Anthropometrics<br>Waist circumference<br>Hip circumference	(mean/SD [‡])	102(15.0)<br>115.1(12.2)	100.2(14.4)<br>115.3(11.8)	104.2(15.5)<br>114.9(12.8)	90.9(16.6)<br>110.6(15.0)
BMI[2]<br>BMI diff<br>Weight diff	(median/IQR[†])	32.8(29.1–39.2)<br>-1.7(-3.7–0.3)<br>-3.6(-9.6–0.3)	31.9(28.8–36.1)<br>-1.5(-3.7–1.3)<br>-1.85(-9.6–3.3)	33.8(29.5–39.7)<br>-2.08(-3.2-(-0.56)<br>-5.2(-8.5-(-1.4)	29.2(24.2–33.4)<br>0.45(-2.7–3.3)<br>-0.4(-8.7–10)
Glucose profiles<br>Fasting glucose	(median /IQR[†])	n = 102<br>5.9(5.1–9.0)	n = 58<br>4.9(4.6–5.2)	n = 44<br>7.5(5.7–11.8)	n = 101<br>4.9(4.6–5.3)
120 mins glucose		n = 78<br>7.1(5.6–9.1)	n = 25<br>5.5(4.8–6.1)	n = 53<br>8.4(6.3–13.2)	n = 99<br>5.5(4.8–6.1)

[†]IQR: Interquartile range

[‡]SD: Standard deviation

[1]GA: Gestational age.

[2]BMI: Body mass index

[3]BP: Blood pressure.

[4]HIV: Human immunodeficiency virus.

[5] OGTT: 75gm 2-hour oral glucose tolerance test

[6]HDP: Hypertensive disorders in pregnancy including gestational hypertension, pre-eclampsia, eclampsia.

[7]OHA: Oral hypoglycaemic agents

[8]LBW: Low birth weight.

[9]SES: Socioeconomic status.

[10]IFG: Impaired fasting glucose.

[11]T2DM: Type 2 diabetes

[12] IGT: Impaired glucose tolerance

[13]FPG: Fasting plasma glucose.

https://doi.org/10.1371/journal.pone.0263529.t001

higher parity, family history of T2DM, and positive HIV status were significant in the distal model. Prior HFDP was significant in the intermediate model, with absence of exclusive breastfeeding being significant in the proximal model. However, in the combined final model only prior history of HFDP, a family history of T2DM and an elevated VAT:SAT ratio were independently associated with risk of progression to T2DM.

iii) Carotid intima media thickness (Table 7). Linear regression models examining maternal factors associated with log cIMT in the various models are displayed in Table 7. These analyses show that of the variables included in the distal model, no variables were significant. In the intermediate model; maternal age, initial SBP remained significant with triglyceride levels and BMI difference being significant in the distal model. In the combined final model, maternal age and SBP at pregnancy and BMI difference were significantly associated with cIMT thickness.

HIV influence on outcomes

The prevalence of HIV within our cohort was 21.6%(n = 44), of which 18.5%(n = 19) were HIV reactive in the HFDP exposed group. The independent influence of HIV on the measured

Table 2. Stratified analysis of maternal outcomes by HFDP groups.

Outcomes at follow-up		HFDP group			Non-HFDP group	p-value
		Total group n = 103	GDM n = 58	DIP n = 45	n = 101	
**Diabetes status**						
Type 2 diabetes mellitus	n(%)	46(44.6)	16(27.6)	30(66.7)	5(4.9)	*[a]0.0001*[b]0.016
Status unknown		27(57.4)	13(81.2)	14(45.2)	4(100)	*[a]<0.001*[b]<0.001
					14(13.9)	*[a]<0.001 *[b]<0.001
Dysglycaemia		57(55.9)	23(39.6)	34(77.3)	69(68.3)	*[a]<0.001 *[b]<0.001
Insulin resistance		92(90.2)	50(87.7)	42(93.3)		
**Cardiovascular**						
Cardiovascular risk factors						
**Historical**						
1.Age	median(IQR)	37.3(33.1–42.7)	37.1(33.9–41.7)	38.1(32.8–42.7)	34.0(29.2–37.3)	*[a] <0.001*[b]0.001
2.Smoking						
Smoker-current/ever	n(%)	10(9.7)	6(10.3)	4(8.9)	8(7.9)	#[a]0.65 #[b]0.901
3. Family history CVD[1]		20(19.4)	10(17.2)	10(22.2)	17(17.1)	[a]0.65. [b]0.90
**Blood pressure**	n(%)	27(26.1)	10(17.2)		12(11.9)	
Chronic HTN[2] ≥ 140/90		22(21)	8(13.8)	17(37.8)	11(10.9)	*[a]0.009 *[b]0.001
Known HTN		5(4.8)	2(3.5)	14(31.1)	1(0.9)	*[a]0.03*[b]0.005
Newly diagnosed				3(6.7)		
		85(82.5)	46(79.3)		73(72.2)	
**Biochemistry**	n(%)	0.8(0.6-.1.14)	0.79(0.6–1.1)		0.58 (0.44–0.8)	*#[a]0.02 *#[b]0.005
1. Proportion with dyslipidaemia	median(IQR)	1.1(0.9–1.3)	1.1(0.9–1.3)	39(86.7)	1.1(0.91–1.36)	[a]0.080*[b]0.146
2. Triglycerides		4.2(3.6–4.8)	4.2(3.6–4.8)	0.9(0.6–1.2)	3.6(3.1–4.4)	*[a]0.000*[b]<0.001
3. High density lipoprotein		2.7(2.1–3.1)	2.7(1.9–3.2)	1.1(1–1.2)	2.2(1.7–2.7)	[a]0.894 *[b]0.8770
4. Total cholesterol				4.2(3.8–4.8)		*[a]0.002*[b]0.002
5. Low density lipoprotein		n = 100	n = 57	2.7(2.2–3.1)	n = 101	[a]0.003*[b]0.002
	median(IQR)					
Cardiovascular risk		8.46(4.9–14.4)	7.17(4.4–10.7)	n = 43	3.48(2.1–5.7)	
a) Framingham risk score	n(%)					*[a]<0.001 *[b]<0.001
(FRS)		13(13)	5(8.7)	11.7(5.8–16.7)	0	
Categories	median(IQR)	29(29)	11(19.3)		8(7.9)	*[a]<0.001 *[b]<0.001
• High risk(>20%)		58(58)	41(71.9)	8(18.6)	93(92.1)	
• Intermediate(10–20%)				18(41.8)		
• Low(<10%)		43.9(25>30–62.8)	35.1(21.1–48.9)	17(239.5)	20.7(12.0–32.7)	*[a]<0.001 *[b]<0.001
	n(%)					
Non-lab based FRS score				53.2(35.5–70.2)		
	median(IQR)					
b) Carotid intima media		0	0	0	0	--
thickness(cIMT)						
• Thickened IMT (>0.8mm)						
• Overall mean CIMT		0.48(0.44–0.53)	0.48(0.44–0.52)	0.49(0.43–0.55)	0.46(0.42–0.50)	*[a]0.0371 [b]0.113
• Right CCA[6]		0.47(0.43–0.51)	0.47(0.42–0.52)	0.46(0.42–0.55)	0.46 (0.42–0.51)	[a]0.35 [b]0.590
• Left CCA		0.49(0.44–0.54)	0.5(0.45–0.54)	0.49(0.44–0.55)	0.46(0.43–0.51)	*[a]0.007*[b]0.0274
**3. Metabolic syndrome**						
Metabolic Syndrome						
Harmonized criteria	n(%)	42(40.8)	17(29.3)	25(55.6)	6(5.9)	[a]<0.001 *[b]<0.001
BMI[3] ≥ 30	n(%)	71(68.9)	39(67.2)	32(71.1)	46(45.5)	*[a] <0.001*[b]<0.003
Waist circumference ≥ 80cm		95(92.2)	54(93.1)	41(91.1)	77(77)	*[a]<0.001 *[b]<0.001
Waist: hip ratio (>0.85)		66(64)	33(56.9)	33(73.3)	26(26)	*[a]<0.001 *[b]<0.010

[a]p-value for control vs. hyperglycemia detected in pregnancy;

[b]p-value for control vs GDM and DIP.

*indicates significance <0.05.

# indicates chi exact test.

†IQR: Interquartile range

‡SD: Standard deviation [1]CVD: Cardiovascular disease.

[2] HTN: Hypertension.

[3] BMI: Body mass index.

[4] CCA: Common carotid artery.

https://doi.org/10.1371/journal.pone.0263529.t002

Table 3. Maternal body composition outcomes by exposure to HFDP vs. non-exposure to HFDP group.

Follow up		HFDP group			Non-HFDP group	p-value
		Total n = 99	GDM n = 55	DIP n = 44	n = 96	
Fat-free soft tissue mass (FFSM)/kg	median (IQR)	44.9(39.2–51.0)	44.1(38.7–49.2)	45.5(41.6–53.7)	41.9(36.2–46.1)	* [a]0.0006*[b]0.0007
Total body fat mass/kg	mean(SD)	36.1(9.6)	34.9(9.2)	37.5(10.1)	31.1(10.7)	*[a] 0.0008 *[b] 0.0011
Total lean body mass/kg	mean(SD)	48.1(7.2)	46.9(6.2)	49.7(8.3)	44.1(6.9)	*[a] 0.0001 *[b] 0.0005
Total body fat %		44.1(40–46.1)	44.3(40.2–47.1)	43.7(40.3–45.3)	43.5(38.9–46.9)	[a]0.42 [b] 0.6146
Android fat/kg	median (IQR)	2.6(2.0–3.4)	2.4(1.9–3.4)	2.9(2.1–3.7)	1.9(1.3–2.7)	* [a]<0.001 *[b] 0.0001
Visceral fat/kg	median (IQR)	1.5(1.2–1.9)	1.3(1.2–1.9)	1.6(1.3–2.0)	1.1(0.7–1.6)	*[a]<0.001 *[b]0.0001
Visceral fat %	median (IQR)	39.8(35–43.6)	38.4(35.4–43.6)	40.2(35.8–43.2)	36.5(29.4–42.5)	*[a] 0.0066 *[b]0.0237
Arm(right) fat mass/kg	median (IQR)	2.1(1.8–2.7)	2.1(1.8–2.6)	2.4(2.0–2.9)	1.8(1.3–2.2)	* [a] <0.001 *[b]0.0001
Leg(right) fat mass/kg	median (IQR)	7.4(5.7–9.1)	7.4(5.6–9.0)	7.1(5.8–8.9)	6.9(5.2–8.5)	[a] 0.13 [b]0.3178
VAT[1] (volume) cm^3	median (IQR)	517.6(372.6–610.2)	510.6(361–594)	570.5(407.9–655.5)	322(219.2–469.2)	* [a]<0.001 *[b] 0.0001
SAT[2](volume) cm^3	median (IQR)	2334.6 (2005.1–3003.6)	2316.2 (1995.4–2949.2)	2492.6 (20240.8–3121.8)	1943.9 (1430–2678.6)	*[a]0.0003 [b]0.0001
**Ratios**		**HFDP group**	GDM	DIP	**Non-HFDP group**	**p-value**
VAT:SAT		0.20(0.16–0.24)	0.20(0.15–0.24)	0.21(0.17–0.24)	0.16(0.12–0.20)	*<0.001
Android: Gynoid		0.93(0.86–1.02)	0.93(0.85–1.02)	0.98(0.86–1.02)	0.86 (0.75–0.95)	[a]*<0.001
Trunk: limb fat mass		0.82(0.73–0.98)	0.81(0.70–0.94)	0.86(0.76–1.01)	0.72(0.60–0.83)	[a]*<0.001
FMI[3] (Fat mass/$height^2$)		13.9(11.7–16.3)	13.4(11.3–15.9)	14.6(12.2–18.0)	12.3(8.9–14.6)	[a]*0.0008
Lean mass//$height^2$		18.7(17.1–20.8)	18.4(17.1–20.3)	19.6(17.0–21.8)	17.1(16.7–18.9)	[a]*<0.001

*indicates significance <0.05

[a]p-value for control vs. hyperglycemia detected in pregnancy;

[b]p-value for control vs GDM and DIP

[1]VAT: Visceral adipose tissue.

[2]SAT: Subcutaneous adipose tissue

[3]FMI: Fat mass.

https://doi.org/10.1371/journal.pone.0263529.t003

outcomes assessed in multivariate regression models was not found to be significant between women with a prior history of HFDP vs those without (Tables 5–7).

Discussion

We found that Black African women with compared to those without a history of HFDP have a (a) 10.5-fold increased risk for developing T2DM(4.6 and 27.6-fold for GDM and DIP groups respectively) (b) 6-fold increased risk of having MetS together with higher visceral adiposity; and (c) higher cardiovascular risk. HIV infection did not influence any of these outcomes.

The rate of progression to T2DM we found was similar to that reported from Cape Town (48% at 5–6 years post-partum) in predominantly mixed-ancestral women using the same diagnostic criteria [7]. This is over 3-fold higher than the background prevalence rates of T2DM for black South African women [30], and identifies a highly vulnerable population.

Table 4. Frequency of the outcome's diabetes, metabolic syndrome and CVD event as predicted by FRS and the adjusted odds ratio for the association with a history of HFDP.

	Diabetes			Metabolic syndrome			Predicted cardiovascular risk (FRS)[b]		
	Crude OR[a] (95% CI)	Adjusted OR[#] (95% CI)	p-value	Crude OR[a] (95% CI)	Adjusted OR[†] (95% CI)	p-value	Crude OR[a] (95% CI)	Adjusted OR[‡] (95% CI)	p-value
HFDP	15.5(5.8–41.3)	10.5(3.7–29.5)	*<0.001	10.9(4.4–27.9)	6.3(2.2–18.1)	<0.001	8.4(3.6–19.1)	4.3(1.6–11.5)	*0.003
GDM	7.3(2.5–21.3)	4.6(1.5–14.4)	0.008	6.5(2.4–17.8)	3.9(1.2–12.5)	*0.021	4.5(1.7–11.4)	1.7(0.52–5.55)	0.373
DIP	38.4(12.9–114.4)	27.6(8.7–87.4)	*<0.001	19.7(7.2–54.5)	10.5(3.3–32.8)	*<0.001	17.7(6.9–45.7)	1.5(0.23–10.0)	*<0.001

[a] p-values for all unadjusted OR was p <0.001.

[b] FRS categorized as intermediate and high (10-year risk of >10% risk for CVD event).

[#]Confounders: Post-partum variables: Maternal age, BMI, Parity and family history diabetes.

[†]Confounders: Index pregnancy variables: Maternal age, parity, SBP, BMI.

[‡]Confounders: Index pregnancy variables: Maternal age, SBP, BMI.

https://doi.org/10.1371/journal.pone.0263529.t004

This risk was significantly higher both in women with GDM, the less severe form of dysglycaemia in pregnancy, as well as women with DIP. Comparisons of our findings with international studies are challenging, as different study designs, lengths of follow-up, definitions and diagnostic criteria are employed. Nevertheless, a recent meta-analysis [31] identified the time period with the highest risk of progression as 3 to 6 years following the pregnancy, which aligns with our findings.

The modifiable risk factors associated with progression and risk of progression to T2DM have best been explored in high income countries(HIC) [32–34] with limited data for low to middle-income countries(LMIC) and very little African representation [35]. Identification of these factors play a key role when implementing strategies to delay or prevent the onset of T2DM. The post-partum period offers a critical window of opportunity to implement such strategies and screen for diabetes in these high-risk individuals. In our study, though a history of HFDP and family history of T2DM and visceral adiposity were significantly associated with progression to T2DM, only adiposity is modifiable. Though these factors have been associated with progression in previous studies, two other studies in South Africa found that a range of measures of the extent of dysglycaemia to be significant predictors: fasting and 2-hr plasma glucose on OGTT in pregnancy, diagnosis of HFDP before 24 weeks indicative of undiagnosed/unrecognized pregestational diabetes, and exposure to insulin and OHA in pregnancy [7,9], all of which are non-modifiable. The protective role of breastfeeding for T2DM [36], was not found to be significant in our study. The high number of women with HFDP who were unaware of their diabetes status indicates that postnatal assessment for diabetes is necessary even in an already overwhelmed LMIC health infrastructure.

Our study highlighted that, in addition to the high risk of progression to T2DM, women with a history of HFDP are more vulnerable to CVD as evidenced by their higher CVD risk scores and greater cIMT. Though their risk scores remained significantly elevated after correcting for obvious confounders, their cIMT, a surrogate marker for identifying pre-clinical atherosclerosis and hence often used as a surrogate CVD measure [37] was independent of prior HFDP and rather influenced by age, SBP and BMI difference. Though no cardiovascular events were reported in our study, their higher cardiovascular risk is likely to translate into CVD events with time as our cohort was young with a relatively short period of follow-up. Consequently, our study did not corroborate the findings of a recent metanalysis [2] demonstrating that women exposed to HFDP have a two-fold higher risk of cardiovascular events independent of the development of T2DM 10–25 years post-delivery.

Table 5. Linear regression model for log-transformed FMI.

Outcome: Fat mass index	M1: Distal model		M2: Intermediate model		M3: Proximal model		M4:M1+M2+M3	
	β (95% CI)	p value	β (95% CI)	p value	β (95% CI)	p value	β (95% CI)	p value
**Maternal pre-pregnancy factors**								
Parity-multiparous	0.18(0.07–0.29)	**0.001**						
Education: secondary and higher	0.03(-0.46–0.53)	0.890						
SES[1] score	0.004 (-0.02–0.03)	0.718						
Smoking	-0.04(-0.21–0.13)	0.654						
Alcohol	0.08(-0.04–0.21)	0.182						
HIV[2] positive	-0.04(-0.16–0.07)	0.476						
**Maternal pregnancy factors**								
Age			-0.001(-0.008–0.005)	0.748				
Initial BMI[3]			0.031(0.02–0.03)	<**0.001**				
Prior HFDP[4]			0.019(-0.07–0.11)	0.683				
**Maternal post-partum factors**								
Exclusive breastfeeding					-0.086(-0.27–0.10)	0.374		
BMI difference[#]					0.009(0.003–0.016)	**0.005**		
Exercise					-0.086(-0.224–0.062)	0.266		
**Combined models**								
Parity							0.023(-0.065–0.112)	0.599
Education: secondary and up							0.167(-0.139–0.474)	0.283
SES score							0.002(-0.015–0.020)	0.818
Smoking							0.053(-0.066–0.172)	0.379
Alcohol							0.001(-0.892–0.091)	0.976
HIV positive							0.092(-0.012–0.198)	0.082
Age							-0.000(-0.006–0.006)	0.982
Initial BMI							0.045(0.039–0.051)	<**0.001***0.539
Prior HFDP							0.029(-0.06–0.124)	0.581
Exclusive breastfeeding							0.033(-0.085–0.151)	<**0.001***0.527
BMI difference							0.043(0.034–0.051)	
Exercise							-0.030(-0.126–0.065)	
	Adj. R^2 = 0.02 N = 191		Adj R^2 = 0.36 N = 187		Adj. R^2 = 0.04 N = 151		Adj. R^2 = 0.66 N = 144	

[1]SES: Socioeconomic asset score,

[2]HIV: Human immunodeficiency virus,

[3]BMI: Body mass index, [4] [5]HFDP: Hyperglycaemia first detected in pregnancy.

[#]BMI diff: The difference between BMI measured at post-partum visit minus BMI at pregnancy.

Values are standardized regression coefficients (β) with 95% confidence interval, and p-values.

https://doi.org/10.1371/journal.pone.0263529.t005

The rates of obesity were high across both groups during the index pregnancy and follow-up, in keeping with high rates of obesity amongst Black women in SA [30]. However, fat distribution differed in those with and without a history of HFDP; with the former having significantly greater upper body fat distribution, in particular, the visceral area. Visceral adiposity is known to be a strong predictor of diabetes and CVD independent of overall fatness [17,38]. South African studies have demonstrated that visceral depots are generally lower in black African women compared to their white female counterparts, despite the higher insulin resistance and high rate of obesity in this group [30,39,40]. Pregnancies complicated by both obesity and HFDP are known to independently influence immediate and long-term maternal outcomes. In our study, initial BMI at index pregnancy and weight gain post-pregnancy were not significantly associated with progression to diabetes or cardiovascular risk outcomes, but visceral adiposity was a significant predictor of progression to T2DM. A better appreciation of the role of obesity and body composition on our measured cardiometabolic outcomes would have been possible if it weren't for our lack of knowledge relating to fat distribution pre- and during pregnancy and BMI measurement at pre-pregnancy and early post-delivery, known to be

Table 6. Multivariate analysis of factors associated with risk of progression to diabetes.

Outcome Diabetes	M1: Distal model		M2: Intermediate model		M3: Proximal model		M4: M1+M2 +M3	
	OR (95%CI)	p value	OR (95% CI)	p value	OR (95%CI)	P value	OR (95%CI)	P value
**Maternal pre-pregnancy factors**								
Parity	5.14(1.82–14.50)	**0.002**						
Education: secondary and higher	0.20 (0.09–4.31)	0.309						
SES[1] score	0.88(0.74–1.05)	0.180						
Alcohol	0.60(0.211–1.71)	0.341						
Smoking	0.40(0.09–1.68)	0.213						
HIV[2]	0.29(0.108–0.82)	**0.019**						
Family history diabetes	4.67(2.15–10.13)	**<0.001**						
**Maternal pregnancy factors**								
Age			1.01(0.94–1.08)	0.704				
Initial BMI[3]			1.04(0.98–1.09)	0.151				
Initial SBP[4]			0.99(0.96–1.02)	0.886				
Prior HFDP[5]			12.9(4.33–38.45)	**<0.001**				
**Maternal post-partum factors**								
Exclusive breastfeeding					0.141(0.04–0.48)	**0.002**		
Exercise					2.28(0.86–6.05)	0.097		
Progesterone only contraception					1.11 (0.49–2.53)	0.794		
VAT:SAT[6] ratio					10.70(2.53–45.25)	**<0.001**		
**Combined models**								
Parity							0.94(0.19–4.55)	0.944
Education: secondary and up							0.32(0.01–8.50)	0.498
SES score							0.75(0.56–1.01)	0.062
Alcohol							0.79(0.18–3.51)	0.763
Smoking							0.31(0.04–2.07)	0.230
HIV							0.40(0.07–2.29)	0.307
Family history diabetes							4.37(1.80–15.80)	**0.010***
Age							0.96(0.88–1.06)	0.482
Initial BMI							1.03(0.94–1.13)	0.407
Initial SBP							0.99(0.95–1.04)	0.840
Prior HFDP							7.84(1.37–44.6)	**0.020***
Exclusive breastfeeding							0.23(0.05–0.96)	0.057
Exercise							1.00(0.25–4.01)	0.998
Progesterone only contraception							0.96(0.33–2.81)	0.955
VAT:SAT ratio							7.98(1.09–57.94)	**0.004***
	N = 200<br>Adj. R^2 = 0.18		N = 193<br>Adj. R^2 = 0.23		N = 151<br>Adj R^2 = 0.15		N = 142<br>Adj. R^2 = 0.37	

[1]SES: Socioeconomic asset score,

[2]HIV: Human immunodeficiency virus,

[3]BMI: Body mass index,

[4]SBP: Systolic blood pressure,

[5]HFDP: Hyperglycaemia first detected in pregnancy,

[6]VAT:SAT ratio: Visceral adipose tissue: Subcutaneous adipose tissue ratio.

Values are standardized odds ratios (OR) with 95% confidence interval, and p-value.

https://doi.org/10.1371/journal.pone.0263529.t006

independent risk factors for progression to T2DM [30]. It is therefore plausible that our study confirmed a high prevalence of MetS in women with HFDP with a 6-fold increased risk after adjusting for age, parity, BMI and SBP. Overall, altered body composition favouring visceral adiposity, together with the increasing burden of HFDP and its cardiometabolic consequences is the setting for the perfect storm in which the coexistence of these entities may perpetuate a vicious cycle fueling the NCD burden.

Of interest, the offspring for this cohort of women were evaluated for childhood adiposity at 3 to 6 years post-partum in another study [41]. Measurements included various anthropometric measures(BMI) and FMI as measured by deuterium dilution method. Maternal BMI

Table 7. Linear regression model for log-transformed carotid intima media thickness (cIMT).

Outcome CIMT	M1: Distal variables		M2: Intermediate model		M3: Proximal model		M4: M1+M2 +M3	
	β (95% CI)	p value	β (95% CI)	p value	β (95% CI)	p value	β (95% CI)	p value
**Maternal pre-pregnancy factors**								
Parity	0.02(-0.011–0.070)	0.163						
Education: secondary and higher	0.02(-0.175–0.207)	0.868						
SES[1] score	0.003(-0.005–0.013)	0.432						
HIV[2] status	0.035(-0.011–0.081)	0.142						
Smoking	-0.019(-0.085–0.046	0.557						
Family history CVD[3]	0.009(-0.039–0.059)	0.695						
**Maternal pregnancy factors**								
Age			0.01(0.007–0.013)	<**0.001**				
Initial BMI[4]			0.002(-0.002–0.004)	0.075				
Initial SBP[5]			0.001(0.000–0.003)	**0.015**				
Prior HFDP			-0.03(-0.07–0.006)	0.095				
**Maternal post-partum factors**								
Exclusive breastfeeding					-0.01(-0.081–0.06)	0.779		
Exercise					0.008(-0.044–0.06)	0.757		
BMI difference					0.002(0.000–0.004)	**0.048**		
TG[6] level					0.042(0.000–0.085)	**0.047**		
HDL[7] level					-0.02(-0.089–0.037)	0.419		
**Combined models**								
Parity							-0.015(-0.060–0.035)	0.555
Education							-0.011(-0.187–0.165)	0.900
SES score							0.007(-0.002–0.016)	0.146
HIV status							0.041(-0.020–0.103)	0.187
Smoking							0.021(-0.046–0.089)	0.533
Family history CVD							0.000(-0.050–0.050)	0.973
Age							0.009(0.006–0.013)	<**0.001***
Initial BMI							0.002(-0.000–0.006)	0.099
Initial SBP							0.002 (0.000–0.003)	**0.018***
Prior HFDP							-0.014(-0.074–0.045)	0.632
Exclusive breastfeeding							-0.001(-0.069–0.066)	0.969
Exercise							0.016(-0.037–0.071)	0.540
BMI difference							0.006(0.002–0.105)	**0.001***
TG level							0.002(-0.004–0.047)	0.929
HDL level							0.011(-0.051–0.075)	0.712
		N = 197 Adj. R^2 = -0.005		N = 192 Adj. R^2 = 0.23		N = 156 Adj R^2 = 0.02		N = 147 Adj. R^2 = 0.21

[1]SES: Socioeconomic asset score,

[2]HIV: Human immunodeficiency virus

[3] CVD: Cardiovascular disease

[4]BMI: Body mass index,

[5]SBP: Systolic blood pressure,

[6] TG: Triglyceride,

[7]HDL: High density lipoprotein.

Values are standardized regression coefficients (β) with 95% confidence interval, and p-value.

https://doi.org/10.1371/journal.pone.0263529.t007

during pregnancy was found to play a more significant role than maternal hyperglycaemia in relation to the outcome.

Though numerous confounders such as BMI, age and parity were identified in relation to the measured outcomes (T2DM, MetS and overall CVD risk), these were found to be non-significant, however only weight based parameters (initial BMI and post-partum weight gain) and not a history of HFDP significantly influenced FMI.

Strengths of our study were the inclusion of a control group of women with confirmed normoglycaemia on OGTT from the same time period and setting as the HFDP group. The

sample was adequately powered for establishing both the primary and secondary outcomes for our study. The use of DXA to assess maternal body composition at 3–5 years post-partum has not previously been reported from Africa. Lastly, exploring the impact HIV had on these outcomes which was insignificant contributes to the limited existing data.

A major limitation of our study was that this was a single centre study which limits the applicability and generalisability. Further limitations included missing pre-pregnancy BMI values for participants leading to difficulty in exploring this variable as a confounder. Difficulty tracing participants resulted in a small sample size and hence less power for sub-group comparisons in particular within the HFDP group (GDM vs. DIP). Self-reporting bias was a potential problem in the administered questionnaire. Cardiovascular risk and body composition outcomes were not measured in pregnancy for longitudinal comparison. However, these parameters are often influenced by the normal physiological adaptations of pregnancy, making interpretation difficult. The use of FRS to calculate cardiovascular risk in these women may have underestimated their risk since the formula doesn't take into account other unique potential risk factors relating to HFDP such as recurrent HFDP and hypertensive disorders of pregnancy. The time of diabetes diagnosis is unclear as women did not have a 6-week post-partum OGTT, in some cases, HFDP may have never resolved post-partum. The small number of women who were HIV positive may have accounted for the lack of effect it had on the measured outcomes. The lack of data surrounding ART regimes was a further limitation as well as the fact that this was a single centre study.

The ever-growing epidemic of diabetes, obesity, and CVD, particularly amongst LMIC populations, poses a significant public health burden, occurring alongside the burden of chronic infectious diseases. Our study identifies a group of young women at high risk of cardiometabolic outcomes, in whom the postpartum period offers a window of opportunity to implement targeted screening, counseling and lifestyle and/or pharmacologic interventions. Future prospective studies are needed to explore the best timing and impact of these interventions in curtailing the adverse outcomes and improving cardiometabolic health in this vulnerable group of women.

Supporting information

S1 Table. Definitions of maternal and neonatal variables and outcomes.
(DOCX)

S1 Appendix. Questionnaire.
(PDF)

Acknowledgments

The authors thank Chris Hani Baragwanath Academic Hospital and the Department of Endocrinology and Obstetrics for access to hospital records, and the study participants for attending the data collection appointment. I would like to acknowledge Glory Chidumwa for his assistance with statistical analysis of the data set.

Author Contributions

Conceptualization: Veronique Nicolaou, Larske Soepnel, Kenneth Huddle, Kerstin Klipstein-Grobusch, Naomi S. Levitt, Shane A. Norris.

Data curation: Veronique Nicolaou, Larske Soepnel.

Formal analysis: Veronique Nicolaou, Shane A. Norris.

Investigation: Veronique Nicolaou, Larske Soepnel.

Methodology: Veronique Nicolaou.

Project administration: Veronique Nicolaou.

Resources: Veronique Nicolaou, Shane A. Norris.

Software: Veronique Nicolaou.

Supervision: Kenneth Huddle, Kerstin Klipstein-Grobusch, Naomi S. Levitt, Shane A. Norris.

Validation: Veronique Nicolaou.

Visualization: Veronique Nicolaou, Larske Soepnel.

Writing – original draft: Veronique Nicolaou.

Writing – review & editing: Veronique Nicolaou, Larske Soepnel, Kenneth Huddle, Kerstin Klipstein-Grobusch, Naomi S. Levitt, Shane A. Norris.

References

1. Bellamy L, Casas JP, Hingorani AD, Williams D. Type 2 diabetes mellitus after gestational diabetes: a systematic review and meta-analysis. The Lancet [Internet]. 2009 May 23 [cited 2020 Sep 1]; 373 (9677):1773–9. Available from: http://www.thelancet.com/article/S0140673609607315/fulltext. https://doi.org/10.1016/S0140-6736(09)60731-5 PMID: 19465232

2. Kramer CK, Campbell S, Retnakaran R. Gestational diabetes and the risk of cardiovascular disease in women: a systematic review and meta-analysis. Diabetologia. 2019; 62:905–14. https://doi.org/10.1007/s00125-019-4840-2 PMID: 30843102

3. Xu Y, Shen S, Sun L, Yang H, Jin B, Cao X. Metabolic syndrome risk after gestational diabetes: A systematic review and meta-analysis. PLoS ONE. 2014 Jan 31; 9(1). https://doi.org/10.1371/journal.pone.0087863 PMID: 24498216

4. Adam S, Rheeder P. Screening for gestational diabetes mellitus in a South African population: Prevalence, comparison of diagnostic criteria and the role of risk factors. South African Medical Journal [Internet]. 2017 May 24 [cited 2018 Feb 9]; 107(6):523. Available from: http://www.samj.org.za/index.php/samj/article/view/11907. https://doi.org/10.7196/SAMJ.2017.v107i6.12043 PMID: 28604326

5. Macaulay S, Ngobeni M, Dunger DB, Norris SA. The prevalence of gestational diabetes mellitus amongst black South African women is a public health concern. Diabetes Research and Clinical Practice [Internet]. 2018 May [cited 2018 Sep 21]; 139:278–87. Available from: http://www.ncbi.nlm.nih.gov/pubmed/29526682. https://doi.org/10.1016/j.diabres.2018.03.012 PMID: 29526682

6. Mamabolo RL, Alberts M, Levitt NS, Delemarre-Van De Waal HA, Steyn NP. Prevalence of gestational diabetes mellitus and the effect of weight on measures of insulin secretion and insulin resistance in third-trimester pregnant rural women residing in the Central Region of Limpopo Province, South Africa. Diabetic Medicine. 2007; 24(3):233–9. https://doi.org/10.1111/j.1464-5491.2006.02073.x PMID: 17263763

7. Chivese T, Norris SA, Levitt NS. Progression to type 2 diabetes mellitus and associated risk factors after hyperglycemia first detected in pregnancy: A cross-sectional study in Cape Town, South Africa. PLoS Medicine. 2019; 16(9). https://doi.org/10.1371/journal.pmed.1002865 PMID: 31498800

8. Chivese T, Norris SA, Levitt NS. High prevalence of cardiovascular risk factors and insulin resistance 6 years after hyperglycemia first detected in pregnancy in Cape Town, South Africa. BMJ Open Diabetes Research and Care. 2019 Nov 1; 7(1):740. https://doi.org/10.1136/bmjdrc-2019-000740 PMID: 31803480

9. Coetzee A, Mason D, Hall DR, Conradie M. Prevalence and predictive factors of early postpartum diabetes among women with gestational diabetes in a single-center cohort. International Journal of Gynecology and Obstetrics. 2018; 142(1):54–60. https://doi.org/10.1002/ijgo.12494 PMID: 29574853

10. Xiang AH, Li BH, Black MH, Sacks DA, Buchanan TA, Jacobsen SJ, et al. Racial and ethnic disparities in diabetes risk after gestational diabetes mellitus. Diabetologia. 2011; 54(12):3016–21. https://doi.org/10.1007/s00125-011-2330-2 PMID: 22016046

11. Black SA. Diabetes, diversity, and disparity: What do we do with the evidence? [Internet]. Vol. 92, American Journal of Public Health. American Public Health Association Inc.; 2002 [cited 2021 Jun 30]. p. 543–8. Available from: https://pubmed.ncbi.nlm.nih.gov/11919048/. https://doi.org/10.2105/ajph.92.4.543 PMID: 11919048

12. Joubert J, Norman R, Lambert E v., Groenewald P, Schneider M, Bull F, et al. Estimating the burden of disease attributable to physical inactivity in South Africa in 2000. South African Medical Journal. 2007; 97(8):725–31. PMID: 17952230

13. Ferrara A. Increasing prevalence of gestational diabetes mellitus: A public health perspective. Diabetes Care [Internet]. 2007 Jul 1 [cited 2021 Jan 14]; 30(SUPPL. 2):S141–6. Available from: https://care.diabetesjournals.org/content/30/Supplement_2/S141. https://doi.org/10.2337/dc07-s206 PMID: 17596462

14. Bao W, Yeung E, Tobias DK, Hu FB, Vaag AA, Chavarro JE, et al. Long-term risk of type 2 diabetes mellitus in relation to BMI and weight change among women with a history of gestational diabetes mellitus: a prospective cohort study. Diabetologia. 2015 Jun 1; 58(6):1212–9. https://doi.org/10.1007/s00125-015-3537-4 PMID: 25796371

15. Snijder MB, Zimmet PZ, Visser M, Dekker JM, Seidell JC, Shaw JE. Independent and opposite associations of waist and hip circumferences with diabetes, hypertension and dyslipidemia: The AusDiab Study. International Journal of Obesity. 2004; 28(3):402–9. https://doi.org/10.1038/sj.ijo.0802567 PMID: 14724659

16. Mtintsilana A, Micklesfield LK, Chorell E, Olsson T, Goedecke JH. Fat redistribution and accumulation of visceral adipose tissue predicts type 2 diabetes risk in middle-aged black South African women: a 13-year longitudinal study. Nutrition and Diabetes. 2019; 9(1). https://doi.org/10.1038/s41387-019-0079-8 PMID: 30918247

17. Chantler S, Dickie K, Micklesfield LK, Goedecke JH. Longitudinal Changes in Body Fat and Its Distribution in Relation to Cardiometabolic Risk in Black South African Women. Metabolic Syndrome and Related Disorders. 2015; 13(9):381–8. https://doi.org/10.1089/met.2015.0021 PMID: 26313235

18. Prioreschi A, Munthali RJ, Soepnel L, Goldstein JA, Micklesfield LK, Aronoff DM, et al. Incidence and prevalence of type 2 diabetes mellitus with HIV infection in Africa: A systematic review and meta-analysis. BMJ Open. 2017; 7:1–11. https://doi.org/10.1136/bmjopen-2016-013953 PMID: 28360243

19. Soepnel LM, Nicolaou V, Huddle KRL, Klipstein-Grobusch K, Levitt NS, Norris SA. Maternal and neonatal outcomes following a diabetic pregnancy within the context of HIV. International Journal of Gynecology & Obstetrics. 2019 Dec 15; 147(3):404–12. https://doi.org/10.1002/ijgo.12956 PMID: 31479156

20. Guariguata L, Linnenkamp U, Beagley J, Whiting DR, Cho NH. Global estimates of the prevalence of hyperglycaemia in pregnancy. Diabetes Research and Clinical Practice. 2014; 103(2):176–85. https://doi.org/10.1016/j.diabres.2013.11.003 PMID: 24300020

21. Census Questionnaire | Statistics South Africa [Internet]. [cited 2021 Jan 19]. Available from: http://www.statssa.gov.za/?page_id=3852.

22. Micklesfield LK, Goedecke JH, Punyanitya M, Wilson KE, Kelly TL. Dual-energy X-ray performs as well as clinical computed tomography for the measurement of visceral fat. Obesity. 2012 May; 20(5):1109–14. https://doi.org/10.1038/oby.2011.367 PMID: 22240726

23. World Health Organization. Definition and diagnosis of diabetes mellitus and intermediate hyperglycemia Report of a WHO/IDF Consultation. 2006.

24. Touboul PJ, Hennerici MG, Meairs S, Adams H, Amarenco P, Bornstein N, et al. Mannheim carotid intima-media thickness and plaque consensus (2004-2006-2011). Vol. 34, Cerebrovascular Diseases. NIH Public Access; 2012. p. 290–6.

25. Stein JH, Korcarz CE, Hurst RT, Lonn E, Kendall CB, Mohler ER, et al. Use of Carotid Ultrasound to Identify Subclinical Vascular Disease and Evaluate Cardiovascular Disease Risk: A Consensus Statement from the American Society of Echocardiography Carotid Intima-Media Thickness Task Force Endorsed by the Society for Vascular. Vol. 21, Journal of the American Society of Echocardiography. Mosby Inc.; 2008. p. 93–111.

26. Alberti KGMM, Eckel RH, Grundy SM, Zimmet PZ, Cleeman JI, Donato KA, et al. Harmonizing the Metabolic Syndrome A Joint Interim Statement of the International Diabetes Federation Task Force on Epidemiology and Prevention; National Heart, Lung, and Blood Institute; American Heart Association; World Heart Federation; International A. Circulation [Internet]. 2009; 120:1640–5. Available from: http://circ.ahajournals.org. https://doi.org/10.1161/CIRCULATIONAHA.109.192644 PMID: 19805654

27. Framingham Risk Score (2008) | QxMD [Internet]. [cited 2021 Jan 18]. Available from: https://qxmd.com/calculate/calculator_252/framingham-risk-score-2008.

28. Harris PA, Taylor R, Thielke R, Payne J, Gonzalez N, Conde JG. Research electronic data capture (REDCap)—A metadata-driven methdology and workflow process for providing translational research informatics support. J Biomed Inform. 2009; 42(2):377–81. https://doi.org/10.1016/j.jbi.2008.08.010 PMID: 18929686

29. StataCorp. StataCorp. 2013. Stata Statistical Software: Release 13. College Station, TX: StataCorp LP. College Station, TX: StataCorp; 2013.

30. Labadarios D, Shisana O, Rehle T, Simbayi L. SANHANES: A unique survey series in the health landscape. South African Medical Journal [Internet]. 2014; 104(10):675–6. Available from: http://www.scielo.org.za/scielo.php?script=sci_arttext&pid=S0256-95742014001000016&lng=en. https://doi.org/10.7196/samj.8842 PMID: 25363051
31. Song C, Lyu Y, Li C, Liu P, Li J, Ma RC, et al. Long-term risk of diabetes in women at varying durations after gestational diabetes: a systematic review and meta-analysis with more than 2 million women. Obesity Reviews. 2018 Mar 1; 19(3):421–9. https://doi.org/10.1111/obr.12645 PMID: 29266655
32. Inoue H, Ishikawa K, Takeda K, Kobayashi A, Kurita K, Kumagai J, et al. Postpartum risk of diabetes and predictive factors for glucose intolerance in East Asian women with gestational diabetes. Diabetes Research and Clinical Practice [Internet]. 2018 Jun 1 [cited 2020 Sep 1]; 140:1–8. Available from: https://doi.org/10.1016/j.diabres.2018.03.031 PMID: 29596944
33. Damm P, Houshmand-Oeregaard A, Kelstrup L, Lauenborg J, Mathiesen ER, Clausen TD. Gestational diabetes mellitus and long-term consequences for mother and offspring: a view from Denmark. Diabetologica. 2016; 59:1396–1399.
34. Eades CE, Styles M, Leese GP, Cheyne H, Evans JM. Progression from gestational diabetes to type 2 diabetes in one region of Scotland: an observational follow-up study. BMC Pregnancy and Childbirth. 2015; 15(1):1–16.
35. Herath H, Herath R, Wickremasinghe R. Gestational diabetes mellitus and risk of type 2 diabetes 10 years after the index pregnancy in Sri Lankan women-A community based retrospective cohort study. PloS ONE [Internet]. 2017; 12(6):1–14. Available from: https://doi.org/10.1371/journal.pone.0179647 PMID: 28644881
36. Feng L, Xu Q, Hu Z, Pan H. Lactation and progression to type 2 diabetes in patients with gestational diabetes mellitus: A systematic review and meta-analysis of cohort studies. Journal of Diabetes Investigation. 2018; 9(6):1360–9. https://doi.org/10.1111/jdi.12838 PMID: 29575786
37. Willeit P, Tschiderer L, Allara E, Reuber K, Seekircher L, Gao L, et al. Carotid Intima-Media Thickness Progression as Surrogate Marker for Cardiovascular Risk: Meta-Analysis of 119 Clinical Trials Involving 100,667 Patients. Circulation [Internet]. 2020 Jun 17 [cited 2020 Sep 1]; 142(7):621–42. Available from: https://www.ahajournals.org/doi/abs/10.1161/CIRCULATIONAHA.120.046361 PMID: 32546049
38. Freemantle N, Holmes J, Hockey A, Kumar S. How strong is the association between abdominal obesity and the incidence of type 2 diabetes? International Journal of Clinical Practice. 2008; 62(9):1391–6. https://doi.org/10.1111/j.1742-1241.2008.01805.x PMID: 18557792
39. Sumner AE, Micklesfield LK, Ricks M, Tambay A v., Avila NA, Thomas F, et al. Waist circumference, BMI, and visceral adipose tissue in white women and women of African descent. Obesity. 2011 Mar; 19 (3):671–4. https://doi.org/10.1038/oby.2010.201 PMID: 20847732
40. Goedecke JH, Levitt NS, Lambert E v., Utzschneider KM, Faulenbach M v., Dave JA, et al. Differential effects of abdominal adipose tissue distribution on insulin sensitivity in black and white South African women. Obesity. 2009 Aug; 17(8):1506–12. https://doi.org/10.1038/oby.2009.73 PMID: 19300428
41. Soepnel LM, Nicolaou V, Slater C, Chidumwa G, Levitt NS, Klipstein-Grobusch K, et al. Obesity and adiposity of 3- to 6-year-old children born to mothers with hyperglycaemia first detected in pregnancy in an urban South African setting. https://doi.org/101080/0301446020211918245 [Internet]. 2021 [cited 2021 Sep 13]; 48(2):81–92. Available from: https://www.tandfonline.com/doi/abs/10.1080/03014460.2021.1918245.
